# Supplementary figures and images for: Extinction and subsequent updating of innate fear responses to a visual looming stimulus rely on hippocampus-dependent mechanisms
Source: PLoS Biol. 2025 Sep 24;23(9):e3003391. doi: 10.1371/journal.pbio.3003391 (PMC12530516; doi:10.1371/journal.pbio.3003391)

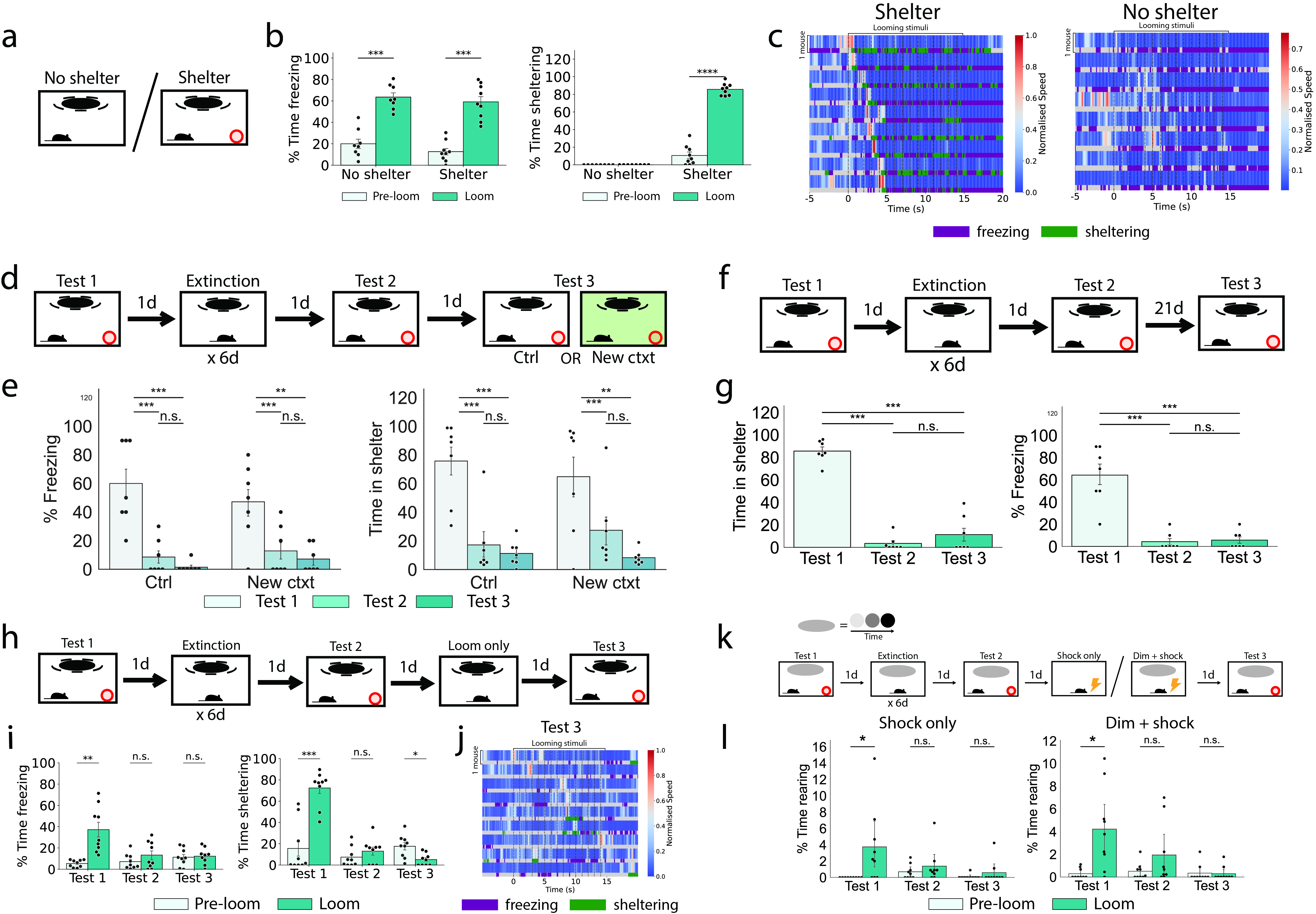

Supplement: S1 Fig — (a) Behavioral paradigm to compare looming responses with and without a shelter. (b) Freezing and sheltering behavior of mice 15 s pre- versus 15 s post-stimulus onset both with and without the presence of a shelter (Student t test; No shelter, n = 8 mice; Shelter, n = 9 mice). (c) Heatmap indicating the speed of mice, with each blue-red row indicating the speed of a single mouse. Beneath the speed, a bar indicates whether the individual mouse was detected as freezing (purple) or sheltering (green). (d) Extinction-renewal assessment paradigm. (e) Freezing and sheltering behavior across test days in the extinction-renewal paradigm (Tukey’s pairwise comparisons; ctrl, n = 7 mice; new context, n = 7 mice). (f) Extinction-spontaneous recovery assessment paradigm. (g) Freezing and sheltering behavior in the extinction-spontaneous recovery paradigm (Tukey’s pairwise comparison; n = 7 mice). (h) Extinction- updating training paradigm with only the looming stimulus. (i) Freezing and sheltering responses of mice from the “loom only” cohort (Repeated measures ANOVA and post hoc Student paired t test; n = 9 mice). (j) Heatmap indicating the speed of mice, with each blue-red row indicating the speed of a single mouse. (j) “Dimming” extinction-updating training paradigm. (k) Rearing responses of mice from the dimming “shock only” and dimming “dim + shock” cohorts in the dimming extinction-updating paradigm (Repeated measures ANOVA and post hoc Student paired t test; n = 9, 9 mice). n.s. p ≥ 0.05, *p < 0.05, **p < 0.01, ***p < 0.001. Details of all statistical comparisons may be found in S1 Data. Underlying raw data may be found in S2 Data. (TIF) [file pbio.3003391.s001.tif]

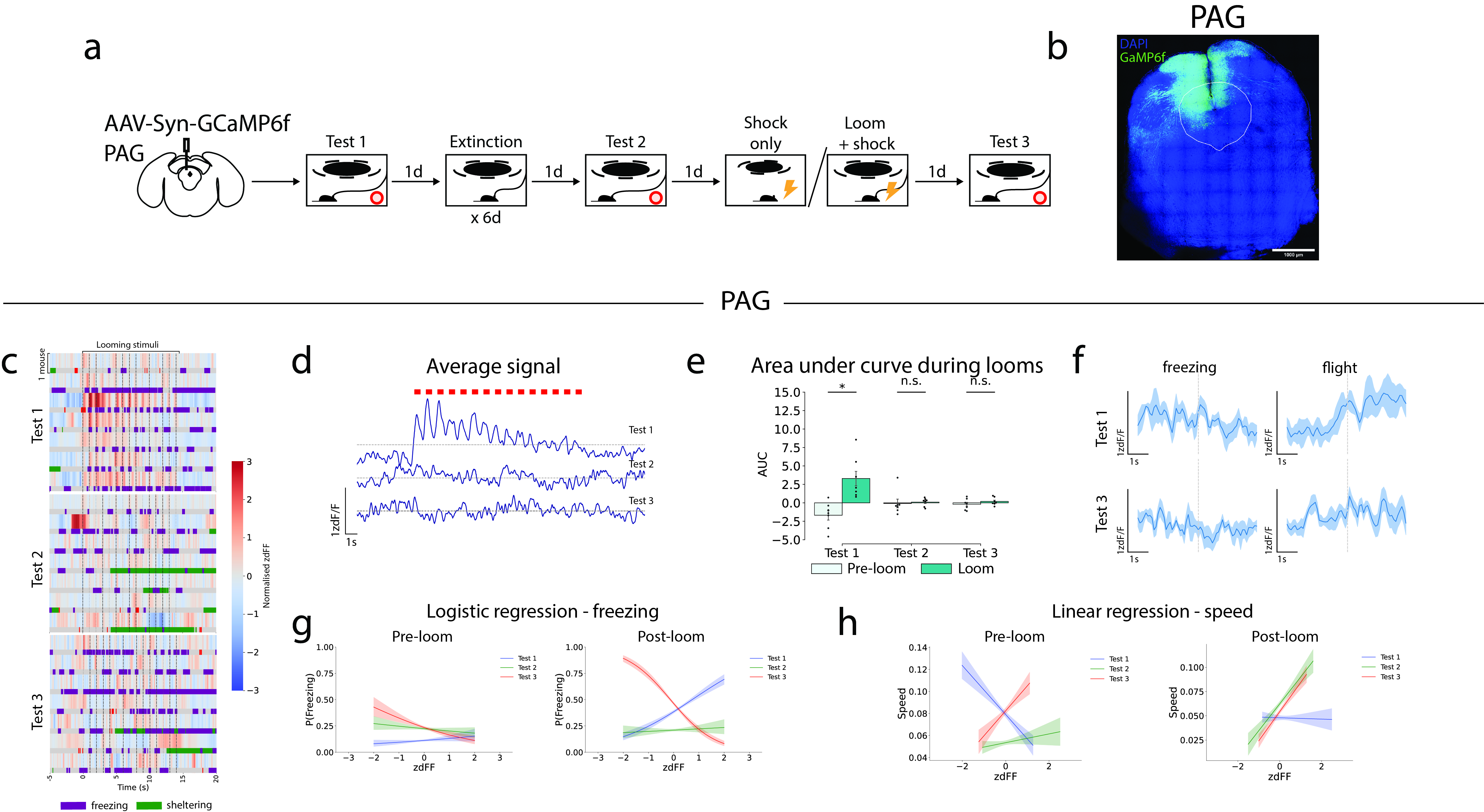

Supplement: S2 Fig — (a) GCaMP was virally expressed in the dorsal PAG. Fiber photometric recordings of GCaMP fluorescence were taken over the course of the extinction-updating training paradigm. (b) Example histology from slices expressing GCaMP in the dorsal PAG. (c) Heatmap with each blue-red row indicating zdFF values from individual mice during looming stimulus presentation. Beneath each zdFF row, a bar indicates whether the individual mouse was detected as freezing (purple) or sheltering (green). Looming stimulus onset is indicated by the dashed black lines, with first stimulus at time = 0 s. Each row is the response from a single mouse. (d) Mean traces of calcium activity in the dorsal PAG on each test day (n = 7 mice). (e) Area under curve of GCaMP signal 15 s pre- and post-stimulus onset, for each day (Student t test). (f) Mean zdFF signal (shaded area = standard error of the mean) across bouts of freezing and flight behavior in all mice on tests 1 and 3. Each behavior is initiated at time = 0 s. (g) Logistic regression of freezing probability with GCaMP signal, 15 s pre- and post-stimulus onset, for each test day. The shaded area is the 95% confidence interval. (h) Linear regression of speed with GCaMP signal, 15 s pre- and post-stimulus onset, for each day. The shaded area is the 95% confidence interval. n.s. p ≥ 0.05, *p < 0.05. Details of all statistical comparisons may be found in S1 Data. Underlying raw data may be found in S2 Data. (TIF) [file pbio.3003391.s002.tif]

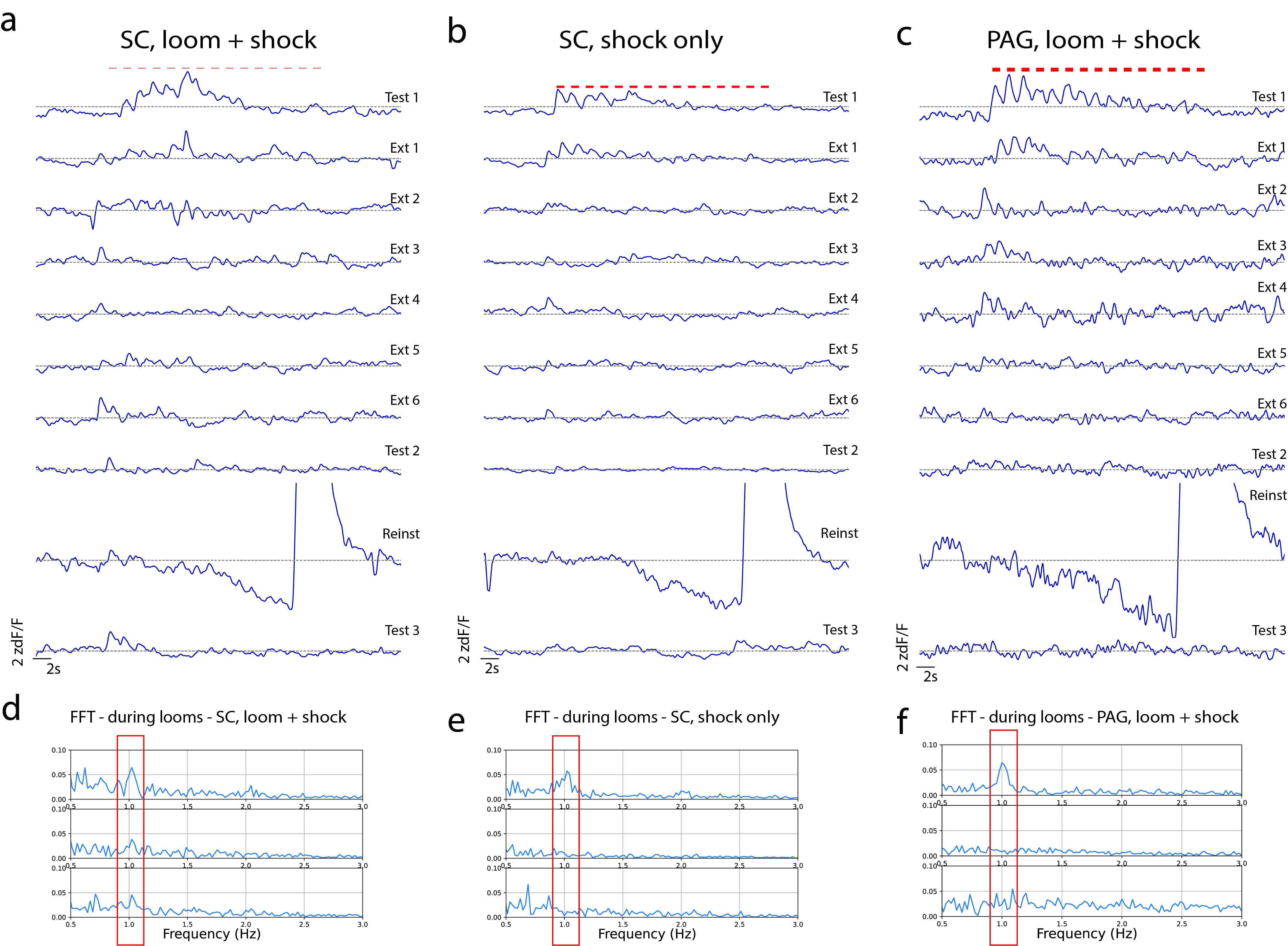

Supplement: S3 Fig — (a) Mean responses of the deep SC across all mice undergoing the loom + shock updating paradigm on each training and test day. (b) As in a, but for mice undergoing the shock-only paradigm. (c) As in a, but with recordings from the dorsal PAG. (d) Fast Fourier transform of the GCaMP signal in the deep SC of “loom ock” mice during stimulus presentation on each day. The most ethologically relevant peak occurs is at 1 Hz, which is the frequency of looming stimulus presentation. (e–f) As in (d), but for (e) the deep SC of “shock only” mice and (f) the dorsal PAG of “loom + shock” mice. (TIF) [file pbio.3003391.s003.tif]

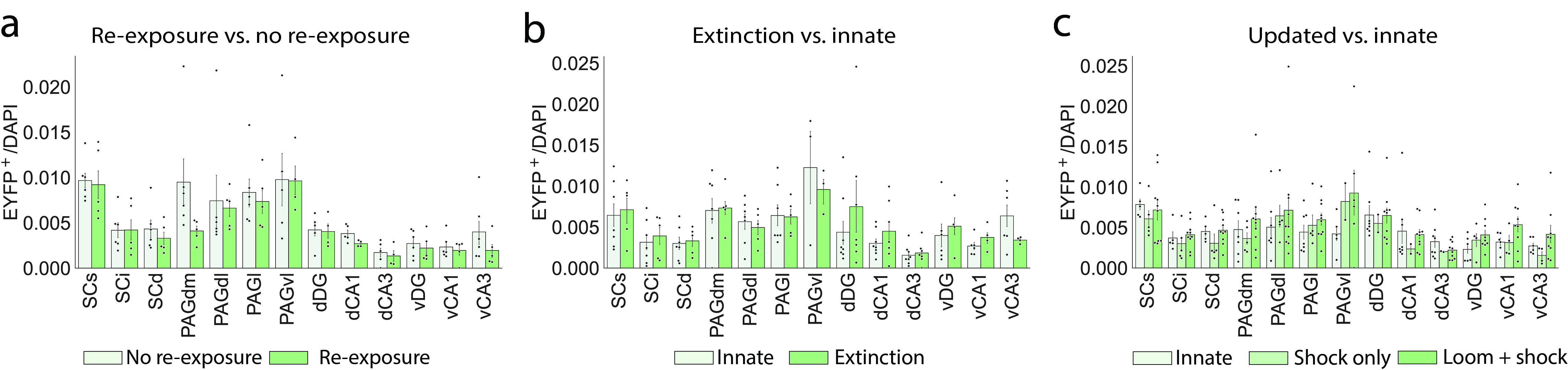

Supplement: S4 Fig — (a) Number of engram cells tagged during looming for the re-exposure (n = 6 mice) vs. no re-exposure (n = 6 mice) paradigm (Student paired t test with False discovery rate correction). (b and c) As in a, but for (b) extinction (n = 7 mice) vs. innate (n = 8 mice), and (c) updating “loom + shock” (n = 11 mice) vs. “shock only” (n = 5 mice) vs. innate (n = 8 mice) (ANOVA with False discovery rate correction). n.s. p ≥ 0.05, #p < 0.05, * q < 0.05; where p is the p value, and q is the false discovery rate-adjusted p value. Details of all statistical comparisons may be found in S1 Data. Underlying raw data may be found in S2 Data. (TIF) [file pbio.3003391.s004.tif]

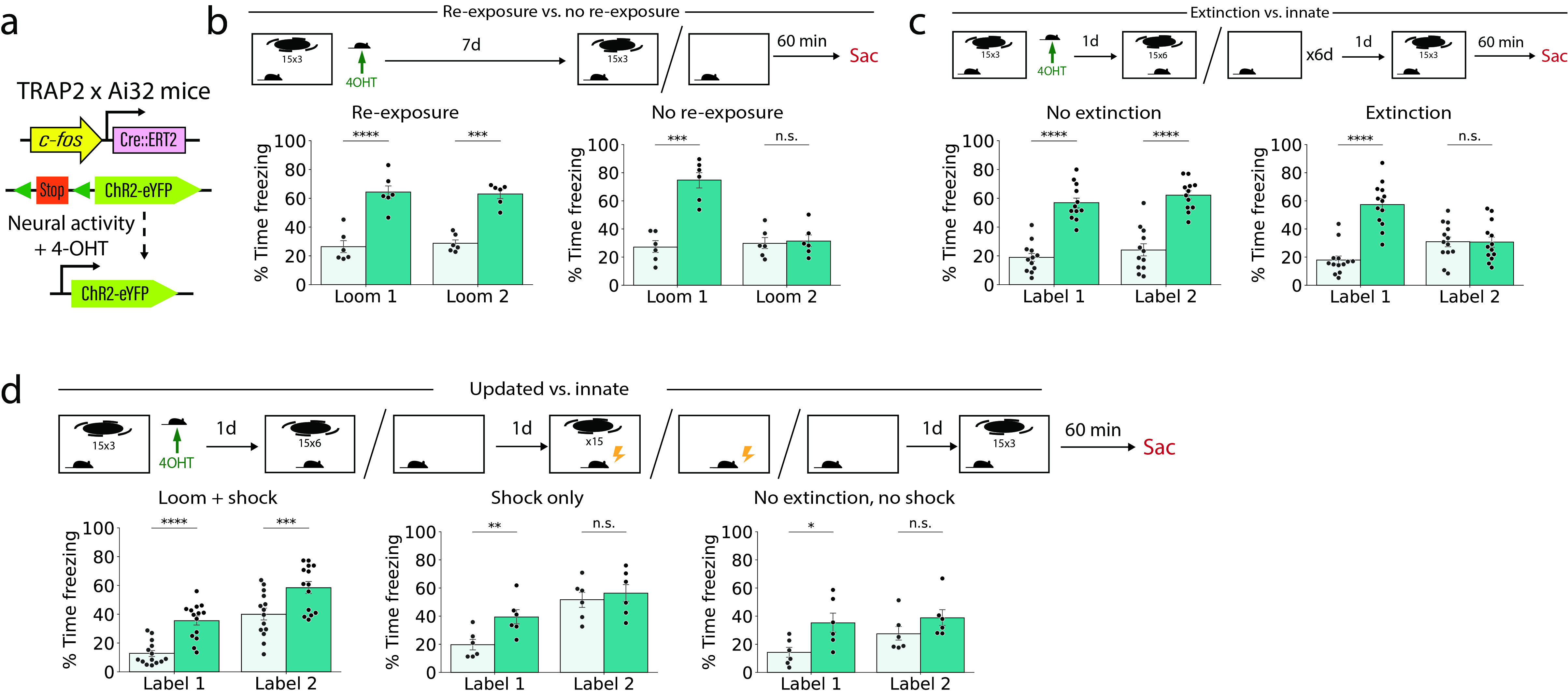

Supplement: S5 Fig — (a) TRAP2xAi32 labeling method. (b) Freezing responses of TRAP2xAi32 mice undergoing the re-exposure or no re-exposure paradigm (Repeated measures ANOVA and post hoc Student paired t test; n = 6, 6 mice). (c) Freezing responses of TRAP2xAi32 mice undergoing the extinction or no-extinction paradigm (Repeated measures ANOVA and post hoc Student paired t test; n = 12, 13 mice). (d) Freezing responses of TRAP2xAi32 mice undergoing the updating “loom + shock”, “shock only” or no-extinction no-shock re-exposure paradigm (Repeated measures ANOVA and post hoc Student paired t test; n = 14, 6, 6 mice). Details of all statistical comparisons may be found in S1 Data. Underlying raw data may be found in S2 Data. (TIF) [file pbio.3003391.s005.tif]

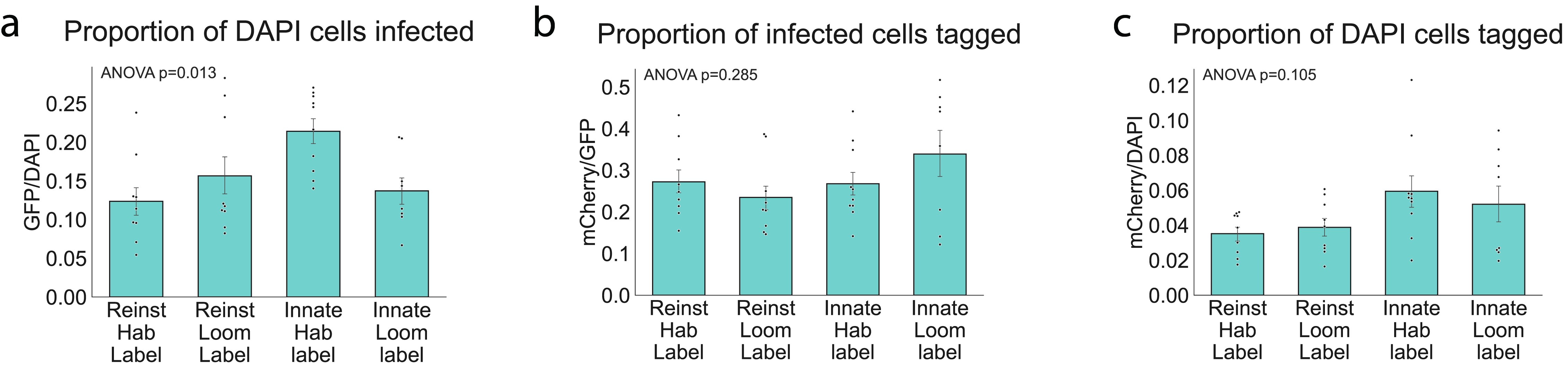

Supplement: S6 Fig — (a) The proportion of DAPI cells which were infected with the f-FLiCRE construct (n = 9, 9, 10, 8 mice). (b) The proportion of infected cells tagged during stimulus presentation (n = 9, 9, 10, 8 mice). (c) The proportion of DAPI cells tagged during stimulus presentation (n = 9, 9, 10, 8 mice). Details of all statistical comparisons may be found in S1 Data. Underlying raw data may be found in S2 Data. (TIF) [file pbio.3003391.s006.tif]
